# Multielements determination and metal transfer investigation in herb medicine Bupleuri Radix by inductively coupled plasma‐mass spectrometry

**DOI:** 10.1002/fsn3.701

**Published:** 2018-09-25

**Authors:** Kunlun Li, Jiaoyang Luo, Tong Ding, Xiaowen Dou, Yuli Hu, Xingguo Zhang, Meihua Yang

**Affiliations:** ^1^ School of Life Science and Engineering Southwest Jiaotong University Chengdu China; ^2^ Institute of Medicinal Plant Development Chinese Academy of Medical Sciences Peking Union Medical College Beijing China

**Keywords:** Bupleuri Radix, decocting, heavy metals, processing, transfer ratios

## Abstract

Bupleuri Radix is a famous traditional Chinese medicine (TCM) and an important raw material in TCM patent prescriptions. It is widely used in several countries, including China, Japan, South Korea, and America. However, the impact of heavy metal transfer rules on TCMs remains unknown. In this study, a total of 45 paired original medicines (OMs), decoction pieces (DPs), and vinegar‐processed (VPs) samples were simultaneously determined via inductively coupled plasma‐mass spectrometry after a microwave digestion. The concentrations of the elements were shown at three levels: (a) Al and Fe at the mg/g level; (b) Pb, Cu, Ba, Mn, Cr, and Ni at the mg/kg level; (c) Co, As, Cd, and Hg at μg/kg level. It is worth noting that the Cu levels were found to exceed the maximum concentration set by Chinese legislation (20.0 mg/kg). In addition, Mn, Ni, and Cu levels were higher in samples from the Gansu province than those from other provinces. The accumulation of the heavy metals decreased in the order of OMs > DPs > VPs; this was especially true for the Al and Fe levels. Furthermore, the results indicate that decocting the samples may reduce the intake of heavy metals. The element transfer ratios for decoctions were under 50% compared to herbal medicines and decreased in the order of Co > As > Mn > Hg > other metals. Our study strongly suggests that long‐term and regular monitoring for heavy metals in the plant is necessary.

## 1 INTRODUCTION

The recent worldwide boom in the popularity of natural herbal medicines has attracted attention to the quality and safety of traditional Chinese medicines (TCMs) (Guo, Wu, Ye, Liu, & Cordell, 2015). Traditionally, the quality and safety control for TCMs is based on its organic compounds (Jiang, David, Tu, & Barbin, 2010). However, the inorganic compounds in TCMs also affect the safety level.

Excess levels of heavy metals are regularly found in rivers and soil, especially in mining and industrial areas (Islam, Ahmed, Raknuzzaman, Habibullah‐Al‐Mamun, & Kundu, 2017; Xiao, Wang, Li, Wang, & Zhang, 2017). Moreover, some vegetables (Hu, Huang, Tian, Holm, & Zhang, 2017), meats (Yi, Tang, Yi, Yang, & Zhang, 2017), and fruits (Fang & Zhu, 2014) have a high heavy metal content. Heavy metal pollution also threatens TCMs and their products due to environmental contamination and various anthropogenic pollutants (Imtiaz et al., 2016). The herb materials used in TCMs typically undergo a series of procedures (cleaning, slicing, drying, processing, etc.) prior to clinical use. Thus, TCM quality control should begin in the fields and continue throughout the production process (Guo et al., 2015). In particular, measuring the level of metal elements during this process is very helpful in assessing the quality and safety of TCMs.

Some essential elements such as iron (Fe) and copper (Cu) are closely related to body growth within a certain concentration; iron supplements have been found to increase hemoglobin and ferritin and reduce the prevalence of anemia (Pasricha, Hayes, Kalumba, & Biggs, 2013). However, the long‐term consumption of heavy metals such as mercury (Hg) and lead (Pb) is hazardous to human health and life (Nordberg & Nordberg, 2016). Some researchers suggest that metal ions bind directly to causative amyloidogenic proteins and modulate their aggregation into amyloids; metal ions are considered to be critical to the etiology of neurodegenerative diseases, such as Minamata disease (Charlet et al., 2012). As the food chain is one of the most important human exposure pathways to heavy metals, it is therefore crucial to regulate TCMs (Hu et al., 2017; Jan et al., 2010). The health risk assessment for heavy metals is always evaluated using the total amount of heavy metals. Health authorities have also established some limit and risk assessment methods (WHO, 2007). In addition, the Target Hazard Quotients have been the key in evaluating the exposure and toxicity according to TCM characteristics (ISO, 2015). What is more, the metal transfer investigation method is a new direction in assessing the elements. As it is known, TCMs are generally decocted before being taken by patients. In other words, only the elements that are transferred to decoction liquids maybe absorbed. Several researchers have proven its validity and usefulness (Da, Hauser‐Davis, Suzuki, & Vitória, 2017; Hu et al., 2017; Liao et al., 2014; Zhao, Wang, Zhao, & Xiang, 2016; Zhao, Wei, Shu, Kong, & Yang, 2016), because of this, the metal transfer investigation method was applied in this study to evaluate the safety control of TCMs.

Several techniques are used for element analysis, including flame atomic absorption spectrometry (Rosa et al., 2015), differential pulse anodic stripping voltammetric techniques (Alghamdi, 2010), stripping potentiometry (Clark, Kontoudakis, Barril, Schmidtke, & Scollary, 2016), capillary zone electrophoresis (Suárez‐Luque, Mato, Huidobro, & Simal‐Lozano, 2006), inductively coupled plasma emission spectroscopy (Chung, Kim, Lee, & Kim, 2015), and microwave plasma atomic emission spectroscopy (Nelson et al., 2015). In particular, the kinetic energy discrimination (KED) in collision/reaction cell ICP‐MS is an effective technique to reduce polyatomic ion interferences (Yamada, 2015); this technique was widely used in this study to alleviate polyatomic interferences.

Bupleuri Radix is an important TCM distributed in the Gansu and Shanxi provinces of north China (Tan, Cai, Hu, & Ni, 2008). Its root is commonly used in several countries including Japan, South Korea, and America. Bupleuri Radix, whether used alone or as a part of a combination of medicines (Chaihu‐Shu‐Gan‐San, etc.), has several useful pharmacological effects. These include alleviating ailments such as influenza, dizziness, lung diseases, chronic gastritis, and cancer (ChP., 2015; Qin, Liu, & Yuan, 2013; Zhao, Wang, et al., 2016; Zhao, We, et al., 2016). In addition, several phytochemical studies have shown that active compounds such as saikosaponin, glycosides, flavonoids, and volatile oil can be isolated from *Bupleurum* L. (Zhao, Li, Yue, Zhang, & Dou, 2012). However, there are few reports concerning the influence of pretreating, processing, or administration on the metal elements levels in Bupleuri Radix, investigation into the transfer rule of elements during processing is especially lacking. Thus, measuring the levels of metal elements is very valuable in controlling the safety of Bupleuri Radix.

In order to ensure the safety of TCMs, it is important to understand the accumulation status and transfer ratios of heavy metals in TCMs. The purpose of this study was to discuss the 12 elements in Bupleuri Radix (Aluminum (Al), Chromium (Cr), Manganese (Mn), Iron (Fe), Cobalt (Co), Nickel (Ni), Copper (Cu), Arsenic (As), Cadmium (Cd), Barium (Ba), Mercury (Hg), and Lead (Pb)), the levels of which vary depending on the habitat of the plant. We also discussed the difference in elements between the original medicines (OMs), decoction pieces (DPs), and vinegar‐processed (VPs) samples of the Bupleuri Radix. Finally, the transfer rules were investigated from the TCMs to their decoctions. This research gave an assessment of herbal medicine quality in the processing industry chain.

## 2 MATERIALS AND METHODS

### 2.1 Materials

A total of 45 paired OMs, DPs, and VPs samples of Bupleuri Radix were collected from the Gansu (15) and Shanxi (30) provinces in China. Professor Yao‐Dong Qi (Institute of Medicinal Plant Development, Chinese Academy of Medical Sciences, Peking Union Medical College) authenticated these samples.

The DPs and VPs of Bupleuri Radix were processed according to the Chinese Pharmacopoeia (ChP). Firstly, the excess impurities and residual stems in the original herbs were removed using ultrapure water. Following this, the OMs were soaked with ultrapure water for 6 hr until they completely absorbed the water; the samples were then cut into thick slices with a guillotine and dried at room temperature for the following tests. Both the VPs and DPs were subjected to traditional Chinese processing techniques (named PaoZhi in Chinese). The DPs were soaked with vinegar at a 1:5 ratio for 6 hr until the vinegar was completely absorbed. They were then baked in an electric oven at 100°C for 2.5 hr and stirred every 30 min. The VPs samples were cooled in room temperature until their next use.

### 2.2 Reagents and apparatus

High‐purity concentrated nitric acid (65% HNO_3_, Trace Metal^™^ Grade) was purchased from Fisher Chemical. The ultrapure water (resistivity > 18.2 MΩ•cm) was prepared using the Milli‐Q system (Millipore Corporation, Bedford, MA, USA). All glass and plastic are were soaked overnight or longer in 10% HNO_3_ and repeatedly rinsed with ultrapure water. Individual stock solutions of Al, Cr, Mn, Fe, Co, Ni, Cu, As, Cd, Ba, Hg, and Pb were obtained from the National Center of Analysis and Testing for Nonferrous Metals and Electronic Materials (Beijing, China). The Certified Reference Material (CRM) *Astragalus* (GBW 10028, GSB‐19), was bought from the National Institute of Standards and Technology (Beijing, China); this was used to test the accuracy of the method.

The digestion was carried out using a microwave oven (Speed Wave^TM^ MWS‐3^+^, Berghof). The ICP‐MS system (Thermo Scientific iCAP^™^ Q ICP‐MS© Thermo Fisher Scientific) was used to simultaneously determine the elements. Thermo Scientific (Hanna‐Kunath‐Strasse 11, 28199 Bremen, Germany) supplied the tuning solution (1.0 μg/l) of ^56^Ba, ^83^Bi, ^58^Ce, ^27^Co, ^49^In, ^3^Li, and ^92^U.

### 2.3 Sample preparation

Surface contaminants of the samples are first removed using ultrapure water. The dried samples can be powdered with a stainless steel blender; all of the powder must be able to pass through the No. 2 Pharmacopoeia sieve with a pore size of 850 μm.

The Bupleuri Radix extract was prepared according to a commonly used method for herbal preparation in China. 10.0 g of the herbal materials were weighed into a glass flask. Next, 80 ml of ultrapure water was added into the flask and boiled for 2 hr. This solution was extracted twice. The liquid extracts were mixed, filtered, and concentrated. After that, the extractum was placed in a vacuumed drying oven and dried at the 30°C. The extractum was coded as W‐OMs, W‐DPs, and W‐VPs. The powder of the dry extractum (0.2 g) was digested through the following procedure.

Approximately 0.2 g of sample material (dry weight) was digested with 6 ml of 65% HNO_3_ in a digestion vessel and kept under stable laboratory conditions overnight. The next day, the samples were digested in a closed microwave system. The digestion process then began and the digestion procedure was as follows: (a) 800 W at 160°C for 20 min; (b) 1400 W at 205°C for 15 min; (c) 0 W for 10 min for cooling. After digestion, the digestion solution was diluted with ultrapure water for a total volume of 50 ml.

### 2.4 Standard analysis

A mixed standard solution was formulated with 10% HNO_3_ and stored at 4°C. The concentrations of the 12 elements were determined via (KED mode) ICP‐MS method. The optimal operating conditions for ICP‐MS analysis are provided in Table 1. The “KED mode” was established as a simple and easy approach to operate the cell‐based ICP‐MS, using 100% He gas for interference reduction (Yamada, 2015).

**Table 1:** Instrumental conditions of the ICP‐MS

Parameter	Value
Collision cell mode	KED
The sensitivity of Co	34,250 cps
Co/Clo Ratio	21
RF incident power	1300 W
Plasma argon flow rate	13 L/min
Auxiliary argon flow rate	0.7 L/min
Nebulizer argon flow rate	0.87 L/min
Scanning mode	Peak jump
Resolution	Standard
Dwell time	10 ms
Sweeps	30
Number of readings per replicate	3
Tuning solvent	^56^Ba,^83^Bi,^58^Ce,^27^Co,^49^In,^3^Li,^92^U

The operating ranges of the calibration curve of the elements were chosen according to the elemental abundance in the samples. Additionally, the correlation coefficient of the linearity was represented by the *r* ^2^ values. The limit of detection (LOD) and limit of quantification (LOQ) were analyzed using a blank solvent (*n *= 20); the LOD had an standard deviation (SD) of 3 and the LOQ had an SD of 10 (Filipiak‐Szok, Kurzawa, & Szłyk, 2015). All the test samples were analyzed in triplicate. In addition, a CRM *Astragalus* standard reference material (GBW 10028) was digested and tested using the same method.

### 2.5 Statistical analysis

Normalized processing is a simplified way to evaluate the data. Namely, the dimensioned expression transforms into a dimensionless expression as a scalar (Ibrahim & Darus, 2008).

The data of the tested elements in Bupleuri Radix were presented as the mean (c) ± SD. The transfer ratios (TRs) of elements for different types of Bupleuri Radix were calculated by Equation (1) to identify the transfer characteristics of elements from TCMs to decoctions.

(1) $$\text{TR}=C_{d}/C_{T}$$

Where *C*_d_ is the concentration of the elements in decoctions after drying, and *C*_T_ is the concentration of the elements (on dry weight basis) in the TCMs (with or without processing).

## 3 RESULTS AND DISCUSSION

### 3.1 Method validation

An external standard was added in order to perform quantitative analysis. The quantitative determinations of the 12 tested elements and their decoctions were carried out by a calibration curve of multielements standard solutions in their optimal measurement concentration ranges. The *r* ^*2*^ values shown in Table 2 were higher than 0.998, revealing a good linearity within the operating ranges. Furthermore, the relative standard deviation of the replicates was calculated to evaluate the precision, which was between 0.61 and 3.53%. This indicates a high degree of stability for the apparatus. Moreover, the LODs and LOQs were in the ranges of 0.001–13.1 ng/ml and 0.010–43.8 ng/ml, respectively. Additionally, the accuracy of the method was assessed through a replicate analysis of the standard reference materials (CRM *Astragalus*, GBW 10028); the results are shown in the Table S1. This shows that the ICP‐MS method is a rapid, stable, and sensitive method to analyze the elements, and that it is fully capable of analyzing the TCMs and their decoctions.

**Table 2:** Calibration curves, linear range, LOD and LOQ for 12 elements

Elements	Calibration curves	Ranges (ng/ml)	*r* ^2^	LOD (ng/ml)	LOQ (ng/ml)	Precision (RSD, %)
Al	y = 4.16e^4^x + 4.18e^6^	62.5–1000	0.9999	3.023	10.08	0.61
Fe	y = 1.72e^3^x + 9.07e^4^	31.2–1000	0.9989	2.381	7.936	1.06
Cr	y = 5.94e^4^x + 5.45e^4^	0.48–62.5	0.9993	0.035	0.118	1.25
Mn	y = 8.93e^4^x + 4.14e^6^	62.5–500	0.9997	13.14	43.80	0.76
Co	y = 6.43e^4^x + 3.55e^4^	0.48–500	0.9993	0.001	0.480	1.25
Ni	y = 1.20e^4^x + 3.45e^4^	0.97–250	0.9985	0.213	0.709	1.21
Cu	y = 3.25e^4^x + 1.07e^3^	0.48–125	0.9995	0.039	0.129	0.99
As	y = 9.33e^3^x + 1.60e^3^	0.48–500	0.9999	0.054	0.180	0.88
Cd	y = 1.37e^4^x + 13.4	0.48–250	0.9999	0.004	0.013	1.87
Ba	y = 1.73e^4^x + 7.00e^3^	0.48–125	0.9999	0.116	0.385	1.47
Pb	y = 8.59e^4^x − 2.58e^3^	0.48–500	0.9999	0.043	0.143	1.76
Hg	y = 1.10e^4^x − 3.25e^2^	0.019–10	0.9993	0.003	0.010	3.53

### 3.2 Determination of the elements in real samples

#### 3.2.1 Concentrations of elements in original medicines

The results of the 12 elements in the OMs samples are represented as c ± SD and are listed in Table 3. Three levels of element concentrations were shown: (a) Al and Fe at the mg/g level; (b) Pb, Cu, Ba, Mn, Cr, and Ni at the mg/kg level; (c) Co, As, Cd, and Hg at the μg/kg level. Aside from Cu and Hg, none of the elements in any of the samples exceeded the limits for medicinal plants according to the ChP. (2.0 mg/kg for As, 0.3 mg/kg for Cd, 5.0 mg/kg for Pb, 0.2 mg/kg for Hg, 20 mg/kg for Cu, and 20 mg/kg for total metals as the above) (Chinese National Pharmacopoeia Commission, 2015), WM/T 2‐2004 (Ministry of Foreign Trade and Economic Cooperation of China, 2004) and ISO18664‐2015 (International Organization for Standardization, 2015).

**Table 3:** Contents of the target elements of OMs in different producing places

OMs	Elements	Average (mg/g) ± SD^a^	Elements	Average (mg/kg) ± SD	Elements	Average (μg/kg) ± SD
Gansu	Al	1.96 ± 0.65	Cr	6.52 ± 1.38	Co	820 ± 341
	Fe	3.01 ± 1.03	Mn	107 ± 27.7	As	950 ± 462
			Ni	34.3 ± 21.6	Cd	116 ± 21.5
			Cu	164 ± 66.0	Hg	50.7 ± 12.6
			Ba	72.2 ± 32.9		
			Pb	2.41 ± 1.54		
Shanxi‐1	Al	1.47 ± 0.55	Cr	12.1 ± 5.29	Co	580 ± 213
	Fe	1.89 ± 0.83	Mn	83.7 ± 18.7	As	702 ± 178
			Ni	30.4 ± 8.54	Cd	180 ± 59.0
			Cu	117 ± 23.7	Hg	220 ± 174
			Ba	104 ± 39.8		
			Pb	2.09 ± 0.39		
Shanxi‐2	Al	2.08 ± 0.42	Cr	7.32 ± 5.85	Co	772 ± 120
	Fe	2.41 ± 0.44	Mn	89.0 ± 15.6	As	663 ± 91.5
			Ni	23.9 ± 6.91	Cd	207 ± 24.8*
			Cu	102 ± 8.0	Hg	147 ± 139.8
			Ba	117 ± 4.10		
			Pb	4.03 ± 0.41#		

*Notes*

^a^Standard deviation.

*p < 0.05, compare with the group of Gansu. #*p *< 0.05, compare with the group of shanxi‐1.

For the OMs derived from Gansu, the concentrations of Fe and Al were in the range of 2.08–3.57 mg/g and 1.32–2.71 mg/g, respectively. Furthermore, the Cr content ranged from 5.18 to 7.93 mg/kg, and the mean levels of Mn, Ni, Cu, Ba, and Pb were 107 mg/kg, 34.3 mg/kg, 164 mg/kg, 72.2 mg/kg, and 2.41 mg/kg, respectively. Co and As concentrations were 820 μg/kg and 950 μg/kg, respectively. Lastly, the mean levels of Cd and Hg were 116 μg/kg and 50.7 μg/kg, respectively. Nearly all the elements were found in low quantities in the Gansu OMs, but the Cu content exceeded the standard limit (Chinese National Pharmacopoeia Commission, 2015; International Organization for Standardization, 2015; Ministry of Foreign Trade and Economic Cooperation of China, 2004).

Geographical environments, including the soil, water, and atmospheric environment, are very important factors in determining the element contents of the plant (Cheng et al., 2014). Compared to the other regions, Gansu and Shanxi have significant differences between their geographical environment and climate environment. The Gansu province is located in the Loess Plateau, where the soil is relatively barren. In Shanxi, the coal industry is vigorously developing; air and soil pollution is therefore widespread in the province (Wu, Zhang, Pei, Chen, & Zheng, 2014). As many studies have shown, the difference in geographical environments is an important cause behind element levels in plants (Kaličanin, Velimirović, Arsić, & Đorđević, 2014; Shahid et al., 2017; Wu et al., 2014); the uptake of metal elements by plants is influenced by various factors, including the nature of soil, climate, and agriculture practices (Hu et al., 2017; Imtiaz et al., 2016). The elements (including Mn, Ni, and Cu) in Gansu were shown to be at higher levels than in the Shanxi provinces. For example, the mean content of Mn was calculated to be 107 mg/kg in Gansu, 83.7 mg/kg in Shanxi‐1, and 89.0 mg/kg in Shanxi‐2. Compared to other areas, the OMs in Shanxi‐1 had relatively low element levels aside from Cr (12.1 mg/kg). What's more, we found that there was no significant difference between the Gansu and Shanxi‐1 samples. When compared with element content in the Gansu region, the concentration of Cd in Shanxi‐2 samples showed obviously higher levels (*p *<* *0.05), with a mean content of 116 μg/kg in Gansu and 207 μg/kg in Shanxi‐2. Aside from this, the Pb content in the samples of various Shanxi cities showed a clear regional difference (*p *<* *0.05), with a mean content of 2.09 mg/kg in Shanxi‐1 and 4.03 mg/kg in Shanxi‐2.

#### 3.2.2 Determination of elements in processed Bupleuri Radix

For TCMs, processing is an essential step before clinical use. The purpose of processing is to remove impurities or change the efficacy; the metal element contents may change during the production process as well (Liao et al., 2014; Rasmussen et al., 2017). According to the results for the DPs (Table 4), almost all the levels of the elements were reduced after processing, especially for Al and Fe (reduced by 35.0%–40.0%, *p *<* *0.05, *p *<* *0.01). Furthermore, the Co and As in major samples were reduced in DPs (*p *<* *0.05, *p *<* *0.01). It was inferred that the washing process might remove some of the heavy metals attached to root surfaces. The mean concentrations of heavy metals in the analyzed VPs samples are shown in Table 4; different elements show different patterns of change. The vast majority of the element levels increased in VPs compared to DPs, especially for Hg. The mean Hg content increased by about 31%, but did not exceed the prescribed limit. Taking into account the type of vinegar as well as the frying process, the growth rate for some elements between the DPs samples and VPs samples is acceptable (Drewnowska et al., 2017; Liao et al., 2014). Further research is needed to explore the effects of the type and amount of vinegar on the metal element content.

**Table 4:** Contents of the target elements of DPs and VPs in different producing places

Samples	Places	Elements	Average (mg/g) ± SD^a^	Elements	Average (mg/kg) ± SD	Elements	Average (μg/kg) ± SD
DPs	Gansu	Al	1.21 ± 0.20*	Cr	5.80 ± 5.70	Co	447 ± 73.7*
		Fe	1.81 ± 0.25*	Mn	82.7 ± 10.2	As	613 ± 113
				Ni	9.76 ± 2.76	Cd	151 ± 65.5
				Cu	68.8 ± 11.2	Hg	115 ± 75.5
				Ba	63.8 ± 34.2		
				Pb	2.03 ± 0.38		
	Shanxi‐1	Al	0.66 ± 0.21**	Cr	11.7 ± 5.47	Co	254 ± 83.7**
		Fe	1.05 ± 0.34*	Mn	65.4 ± 12.6	As	354 ± 35.7**
				Ni	17.0 ± 5.25*	Cd	194 ± 58.7
				Cu	69.5 ± 24.3**	Hg	62.7 ± 14.4
				Ba	88.7 ± 49.2		
				Pb	2.65 ± 0.62		
	Shanxi‐2	Al	1.34 ± 0.56	Cr	6.20 ± 6.13	Co	468 ± 72.0*
		Fe	1.63 ± 0.53	Mn	78.3 ± 7.07	As	435 ± 161
				Ni	13.2 ± 4.11	Cd	202 ± 75.9
				Cu	69.0 ± 6.62**	Hg	53.2 ± 5.14
				Ba	112 ± 4.33		
				Pb	3.23 ± 0.52		
VPs	Gansu	Al	0.87 ± 0.18**	Cr	2.70 ± 0.48	Co	396 ± 54.2*
		Fe	1.58 ± 0.24*	Mn	98.1 ± 6.20	As	645 ± 221
				Ni	13.5 ± 4.95	Cd	202 ± 28.4*
				Cu	72.3 ± 6.94	Hg	397 ± 56.4**
				Ba	61.1 ± 32.2		
				Pb	2.93 ± 0.16		
	Shanxi‐1	Al	0.84 ± 0.30*	Cr	7.95 ± 6.18	Co	367 ± 154
		Fe	1.30 ± 0.52	Mn	93.9 ± 22.2	As	472 ± 120*
				Ni	18.2 ± 8.51*	Cd	201 ± 65.7
				Cu	117 ± 23.7*	Hg	544 ± 379
				Ba	104 ± 39.8		
				Pb	2.42 ± 0.30		
	Shanxi‐2	Al	1.42 ± 0.68	Cr	7.34 ± 7.99	Co	565 ± 140
		Fe	1.72 ± 0.74	Mn	108 ± 8.52	As	492 ± 148
				Ni	23.5 ± 7.97	Cd	251 ± 144
				Cu	78.4 ± 13.3*	Hg	156 ± 39.9
				Ba	122 ± .91		
				Pb	2.89 ± 0.94		

*Notes*

^a^Standard deviation.

*p < 0.05, **p < 0.01, compare with the corresponding OMs group.

### 3.3 Regularity research for the elements

#### 3.3.1 Regularity of the element contents in Bupleuri Radix

**Figure 1:**
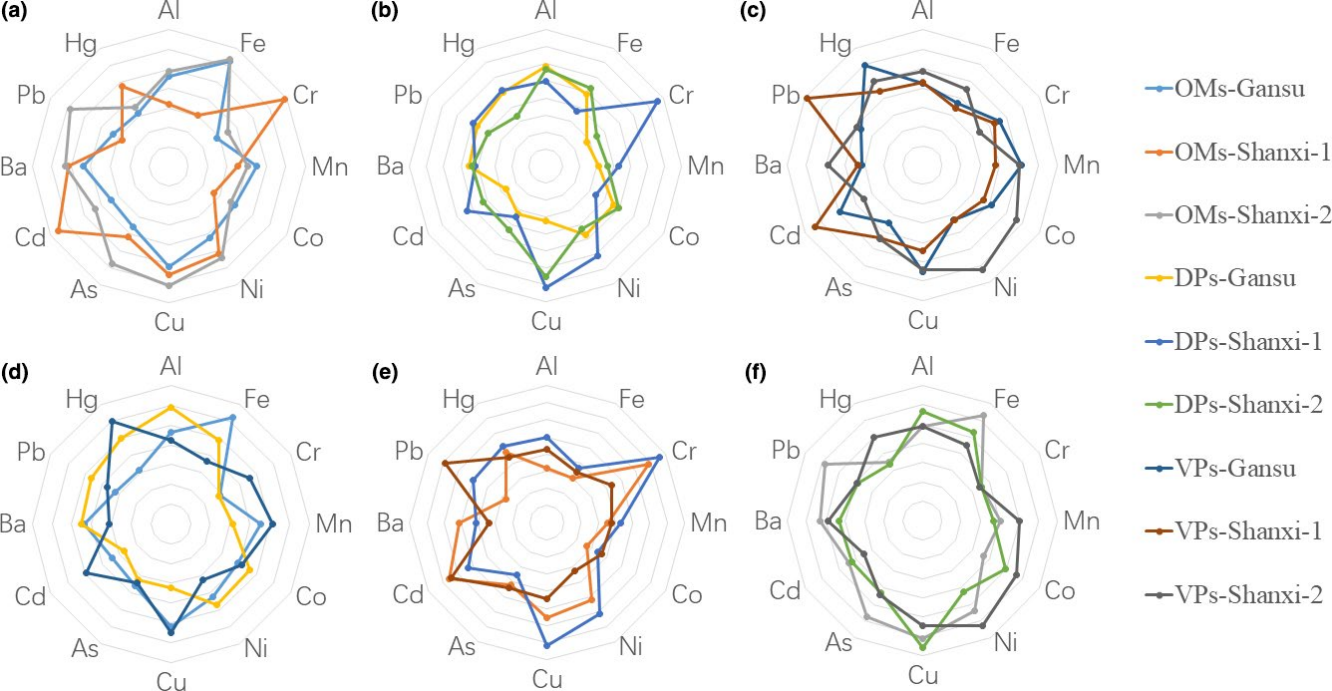
The contents of elements in TCMs among different samples of production places and processing. (a) the relative content of elements in OMs samples among different place; (b) the contents of elements in DPs samples between among production places; (c) the contents of elements in VPs samples among different production places; (d) the contents of elements among different processing samples in Gansu; (e) the contents of elements among different processing samples in Shanxi‐1; (f) the contents of elements among different processing samples in Shanxi‐2

In this section, the levels of metal elements in samples from different places of origin and under different processing methods are discussed. We measured the data by normalization; the relative content of elements in TCMs shown in Figure 1. For the OMs samples (Figure 1a), it was found that different elements had similar laws of variation in the Gansu and Shanxi‐2 production processes and that Cr and Cd had relatively higher element levels in Shanxi‐1. Additionally, the relative content of elements in DPs and VPs samples tended to be stable (Figure 1b,c), especially for Co, Mn, Fe, Al, Hg, and Ba. The stability of their element contents suggests that these elements are less affected by geographical factors, while more sensitive elements (including Cr, Cd, and Pb) exhibit specific regional differences and greater toxicological activity.

Our studies have also indicated that the change in elements is sensitive within OMs, DPs, and VPs. The relative element levels showed that processing could reduce the contents of metals, especially in decoction pieces. According to the results shown in Figure 1d–f, processing led to a decrease in some elements, particularly exogenous elements such as heavy metals. Furthermore, the contents of certain elements increased (e.g., Cd) after the PaoZhi process, but others decreased (e.g., Cr and Cu). This may have been related to the solvent and processing mechanism; however, the main factor behind this phenomenon should be studied in future investigations. Simple processing, such as cleaning, can significantly reduce the contents of heavy metals; meanwhile, complex processing (e.g., PaoZhi) may increase the levels of some elements because of the solvent and processing equipment.

#### 3.3.2 Regularity of the transfer ratios

There is a significant amount of research concerning the transfer of TCMs into decoctions, such as *A*. *oxyphylla* and *M*. *officinalis*. Zhao, Wang, et al. (2016); Zhao, We, et al. (2016) demonstrated that the transfer ratios (TRs) of the investigated elements were different and that it might depend on the types of elements, the plant species, and the extraction methods. In order to ensure safe levels of heavy metals in Bupleuri Radix decoctions, we calculated the transfer ratios of the elements from raw TCMs into the decoctions.

**Figure 2:**
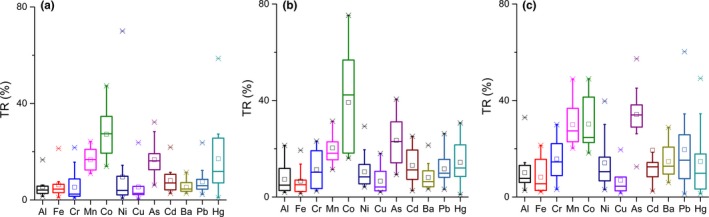
Transfer ratios of elements for different forms of Bupleuri Radix and their decoctions. (a) the transfer ratios of elements of OMs samples and their decoctions; (b) the transfer ratios of elements of DPs samples and their decoctions; (c) the transfer ratios of elements of VPs samples and their decoctions

The test results are shown in Table 5. The mean yields of the W‐OMs, W‐DPs, and W‐VPs samples were 14.2%, 15.5%, and 21.8%, respectively. In addition, the range of the TRs was from 5.29% (Cr) to 27.2% (Co) in the OMs samples, 6.59% (Fe) to 39.2% (Co) in the DPs samples, and 8.23% (Fe) to 34.2% (As) in the VPs samples. For the elements in the three types of Bupleuri Radix, the TRs were low and the contents of heavy metals in the decoctions did not exceed the set limits. The TRs varied significantly, depending on the different elements and processing types (Figure 2). In Figure 2a–c, it was found that the TRs of Mn, Co and As were higher than those of other metals. The TRs of heavy metals for a different method of processing also varied significantly; the VPs samples showed a relatively high transfer rate, except for that of Co and Hg. Decoction is the main medication of TCMs, so we must take precautions as high TRs of heavy metals would be harmful to our health (Cinnirella, Hedgecock & Sprovieri, 2014).

**Table 5:** Transfer ratios of the 12 elements in different batches of Bupleuri Radix

Elements	TR (%)		
	OMs	DPs	VPs
Al	5.44	7.36	10.1
Fe	6.29	6.59	8.23
Cr	5.29	11.3	15.8
Mn	16.7	20.4	30.0
Co	27.2	39.2	30.2
Ni	9.53	10.5	14.1
Cu	5.38	6.60	6.95
As	16.7	23.5	34.2
Cd	8.06	13.1	19.4
Ba	5.30	8.11	14.7
Pb	7.53	11.7	19.7
Hg	17.1	14.3	14.7

**Figure 3:**
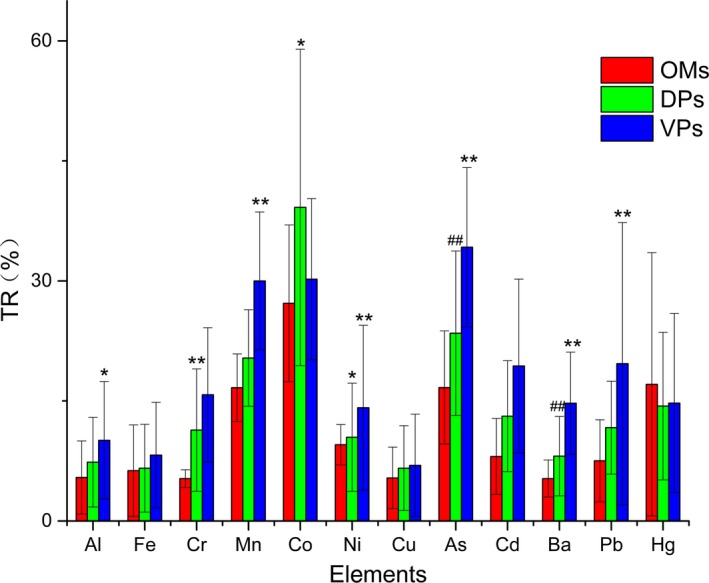
The transfer ratios of elements for different types of samples and their decoction. *p <* *0.05, **p <* *0.01, compare with the OMs group. ^##^ *p *<* *0.01, compare with the VPs group

According to the results in Figure 3, the DPs and VPs samples had relatively higher TRs of heavy metals, aside from Hg. Additionally, the mean TRs of different heavy metals decreased in the order of Co > As > Mn > Hg > other metals. The results also showed that the TRs of all the elements were below 50%, within which 10% of the elements had TRs at <10%. Furthermore, we found that the processing can increase the leaching rate of most elements (*p *<* *0.05, *p *<* *0.01) and can make it easier for the element to transfer into the water solution (Rasmussen et al., 2017). Previous studies have indicated that Co and As could be easily mobilized from the herbs to the decoctions (Fattahi, Rashchi & Abkhoshk, 2016; Obeidy et al., 2016). In this study, we found that Co and As had the highest TRs values, thus the two elements should be closely monitored to prevent further transferring from herbal medicines to decoctions. In terms of the TRs research on heavy metals, water extraction can significantly reduce the contents (Zhao, Wang, et al., 2016; Zhao, We, et al., 2016). The result also points out that the PaoZhi method may help heavy metals in the herb dissolve more easily into its decoction.

## 4 CONCLUSION

In this study, an exclusive, rapid, stable, and sensitive method based on (KED mode) ICP‐MS was applied for the analysis of trace elements, with satisfactory results. A total of 12 elements were determined in 45 paired Bupleuri Radix samples after microwave digestion. On one hand, nearly all the contents of elements decreased after processing. On the other hand, the growth rate of some metals such as Al and Fe in VPs is likely to increase due to material processing.

Additionally, we found that the processing increases the leaching rate of most elements. The DPs and VPs samples had relatively higher TRs of heavy metals compared to those of the OMs, aside from Hg. The mean TRs of different heavy metals was found to decrease in the order of Co > As > Mn > Hg > other metals. Furthermore, the processing (PaoZhi) showed a great influence on the TRs between Bupleuri Radix and its decoctions. The present study suggests that the transfer rules research for elements in medicinal plants can have a significant impact. Thus, there should be long‐term and regular monitoring for heavy metals pollution in plants, with a focus on their processing methods.

## CONFLICT OF INTEREST

The authors declare that there are no conflict of interest.
