# Supplementary material for: Multielements determination and metal transfer investigation in herb medicine Bupleuri Radix by inductively coupled plasma‐mass spectrometry
Source: Food Sci Nutr. 2018 Sep 25;6(8):2005–14. doi: 10.1002/fsn3.701 (PMC6261210; doi:10.1002/fsn3.701)
Supplement: Supplementary file 1 [file FSN3-6-2005-s001.doc]

**Supplementary data**

**Table 1** The degree of coincidence between the standard value and the measured value of CRM *Astragalus*

| Elements | Standard value | Measured value |
| --- | --- | --- |
| Al* | 0.18±0.03 | 0.20±0.01 |
| Fe* | 0.113±0.007 | 0.115±0.005 |
| Cr | 2.2±0.4 | 2.11±0.08 |
| Mn | 33±1 | 33.65±0.23 |
| Co | 0.44±0.03 | 0.44±0.02 |
| Ni | 2.26±0.15 | 2.32±0.08 |
| Cu | 8.5±0.014 | 8.49±0.03 |
| As | 0.57±0.05 | 0.58±0.02 |
| Cd | 0.042±0.01 | 0.041±0.08 |
| Ba | 20.5±2.5 | 19.13±0.13 |
| Pb | 1.44±0.10 | 1.38±0.07 |
| Hg** | (12) | 0.32±0.05 |

* the unit of content is 10^-2^, ** the unit of content is 10^-9^, the datas after "±" is the uncertainty, and the value in parentheses is the reference value.
